# Rapid Fabrication of Continuous Surface Fresnel Microlens Array by Femtosecond Laser Focal Field Engineering

**DOI:** 10.3390/mi11020112

**Published:** 2020-01-21

**Authors:** Linyu Yan, Dong Yang, Qihuang Gong, Yan Li

**Affiliations:** 1 State Key Laboratory for Mesoscopic Physics, Collaborative Innovation Center of Quantum Matter, Department of Physics, Peking University, Beijing 100871, China; yanlinyu@pku.edu.cn (L.Y.); yangdong@pku.edu.cn (D.Y.); qhgong@pku.edu.cn (Q.G.); 2 Frontiers Science Center for Nano-optoelectronics, Peking University, Beijing 100871, China; 3 Collaborative Innovation Center of Extreme Optics, Shanxi University, Taiyuan 030006, China

**Keywords:** femtosecond laser direct writing, two-photon polymerization, three-dimensional focal field engineering, Fresnel lens

## Abstract

Femtosecond laser direct writing through two-photon polymerization has been widely used in precision fabrication of three-dimensional microstructures but is usually time consuming. In this article, we report the rapid fabrication of continuous surface Fresnel lens array through femtosecond laser three-dimensional focal field engineering. Each Fresnel lens is formed by continuous two-photon polymerization of the two-dimensional slices of the whole structure with one-dimensional scan of the corresponding two-dimensional engineered intensity distribution. Moreover, we anneal the lens array to improve its focusing and imaging performance.

## 1. Introduction

Microlens is becoming an indispensable optical element in modern photonic devices. Among several kinds of microlens, benefiting from the advantages of compactness, lightweight construction, high numerical aperture, short-focal length, and excellent focusing capability, the Fresnel lens has found practical applications in integrated optical systems, such as solar distillation concentrator [1], optical multiplexer/demultiplexer [2,3], infrared-laser beam scanning system [4] and fluorescence detector for ultra-high energy cosmic ray observation [5]. Arrays of Fresnel lens have been widely used in diverse optical devices such as scanners, charge-coupled device (CCD) image sensors [6] and pyroelectric infrared sensors [7], they also provide great potential in optical interconnection of large-scale parallel computers [8].

The electron-beam lithography [6], the diamond turning process [9] and the direct writing two-photon polymerization (TPP) [10] have currently been adopted to fabricate the Fresnel lens arrays. Among them, femtosecond laser direct writing TPP, which utilizes high intensity laser-induced two-photon absorption to initiate polymerization, is a potential microstructure fabrication technology with high precision and true three-dimensional (3D) configuration [11]. It has been considered as a promising method for micro-optical devices [12,13,14], microfluidic device, tissue engineering [15,16,17], etc. However, the traditional point-by-point scanning usually takes a long time, thus it is difficult to fabricate large-sized structures or arrays. Therefore, various solutions have been proposed to speed up the process, for example, the focal field engineering [18,19,20,21,22], the multi-focus generation for parallel processing [23,24,25], and the multi-beam interference [26,27] for specific periodic structures. Previous researches have shown that the focal field engineering has great potential in fabricating 3D structures [22].

In this article, we report on the rapid fabrication of continuous surface Fresnel lens array with the 3D focal field engineering to facilitate the fabrication speed of the two-photon polymerization. Furthermore, we anneal the lens array to improve its focusing and imaging performance.

## 2. Materials and Methods

**Figure 1:**
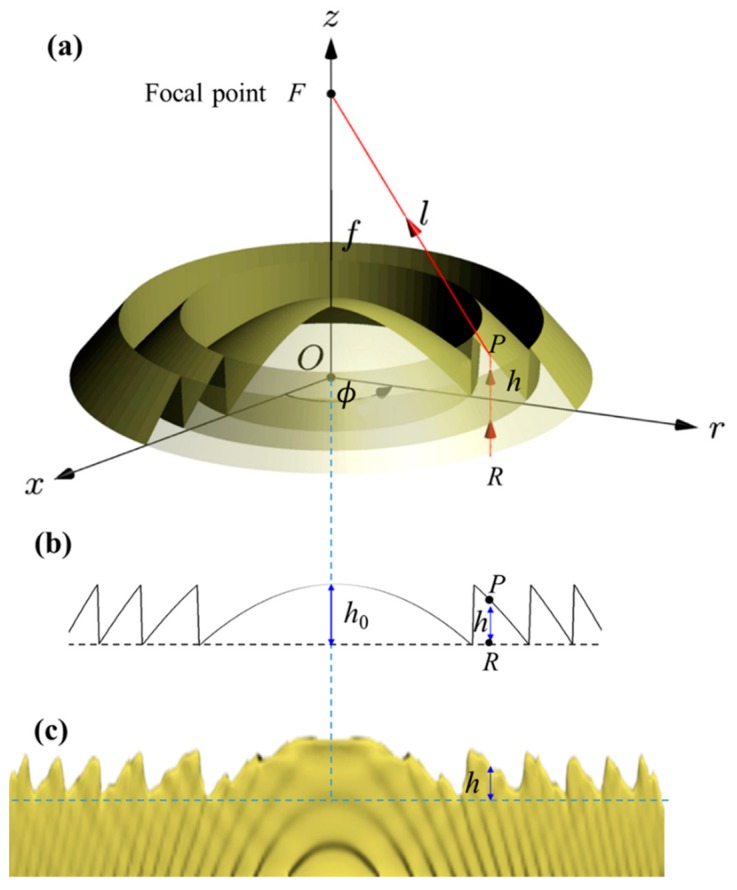
(**a**) Schematic of a continuous surface Fresnel lens; (**b**) cross section of the designed Fresnel lens; (**c**) corresponding slice of the focal filed intensity generated by 3D focal field engineering.

The configuration of the Fresnel lens fabricated in this work with a diameter of 40 µm and a focal length *f* of 50 µm is shown in Figure 1a. Its surface relief profile is presented in Figure 1b. Figure 1c is the corresponding slice of the focal filed intensity generated by 3D focal field engineering. A pedestal with a thickness of 2 µm is added beneath the Fresnel lens to compensate the surface fluctuation or the tilt of the substrate.

For a continuous surface Fresnel lens with the focal length *f*, the optical path length (*l*) from incident point *R*(*r*, *ϕ*, *z* = 0) to the point *P*(*r*, *ϕ*, *z* = *h*) on the surface with the local height *h* then to the focal point *F*(0, 0, *z* = *f*) should satisfy the following equation:

(1) $$l=l_{0}+i\lambda$$

where *i* is the order of Fresnel zone, λ is the wavelength. The optical path length *l*_0_ from *O* to *F* is a constant and *l*_0_ = (*n* − 1)*h*_0_ + *f*. In this work, *h*_0_ = 1.5 µm, λ = 633 nm, the refractive index of the polymer is *n* = 1.516. The corresponding phase change is $\varphi =\varphi_{0}+i\cdot 2\pi$.

According to the coordinates in Figure 1a, *l* can be written as:

(2) $$l=nh+\sqrt{{\left(f-h\right)}^{2}+r^{2}}$$

From Equations (1) and (2), the required height of the Fresnel lens can be expressed as follows:

(3) $$h=\frac{nl-f-\sqrt{\left({\left(nl-f\right)}^{2}-\left(n^{2}-1\right)\left(l^{2}-f^{2}-r^{2}\right)\right)}}{n^{2}-1}$$

**Figure 2:**
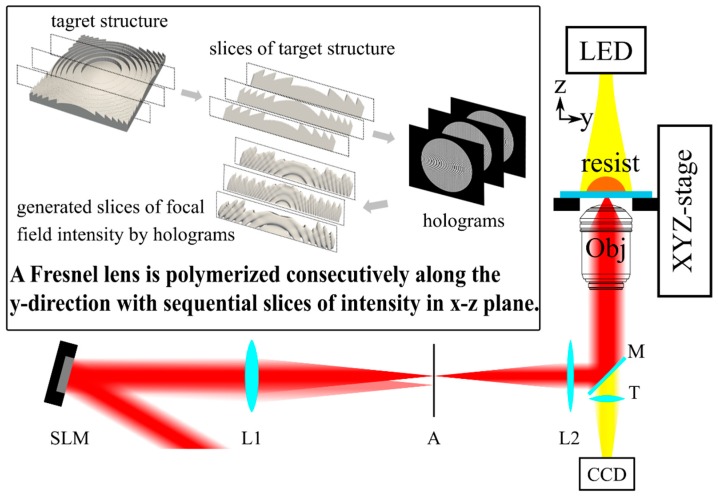
Schematic of the experimental setup for 1D scan two-photon polymerization (TPP) of a Fresnel lens with 3D focal field engineering. SLM: Spatial light modulator; L1 (1000 mm), L2 (400 mm), two lenses of 4f system; A: Aperture; M: Mirrors; LED: Light-emitting diode for illumination; T: Tube lens; Obj: Objective lens; CCD: Charge coupled device. The inset is the flow chart of 2D sliced focal field intensity profiles generated by holograms calculated by the slices of the target structure.

The maximum height of the Fresnel lens in this work is approximately 1.5 µm. Since the imaging performance is highly sensitive to the height distribution of the Fresnel lens, we fabricate the lens by the two-photon polymerization to meet the extreme accuracy requirements, as shown in Figure 2. The excitation source is a femtosecond laser (PH1-20, Light Conversion, Vilnius, Lithuania), which generates 240 fs pulses at 1030 nm, with a repetition rate of 1 MHz. The incident light is modulated by a spatial light modulator (SLM) (X13138-03, Hamamatsu Photonics K.K., Hamamatsu, Japan) and projected by a 4f system onto the pupil of an oil immersion objective lens with a numerical aperture of 1.4 (UPLSAPO 100×, Olympus, Tokyo, Japan). The 4f system consists of two lenses with focal lengths of 1000 mm (L1) and 400 mm (L2), respectively. In order to remove the unwanted diffraction orders by the spatial filtering, an aperture is placed in the 4f system. After modulating, projection, filtering, and focusing, the laser is focused into the resist (SZ2080, FORTH, Heraklion, Greece). A XYZ-stage (FG1000-3D, Aerotech, Pittsburgh, USA) translates the sample to control the relative position of the focal field in the resist. Light-emitting diode (LED) and CCD are used to locate the exposure position and observe the polymerization process.

The entire target structure is fabricated by continuous polymerization of the vertical 2D slices of the structure with 1D scan. First, we divide the Fresnel lens into 200 vertical slices with a thickness of 200 nm and engineer the sliced 2D focal field according to these slices, respectively. Then, the corresponding holograms are calculated via the Gerchberg–Saxton algorithm, similar to the procedure described in Supporting Information in Ref. [22]. We adapt the constraints and the initial conditions of the Gerchberg–Saxton algorithm for the 3D focal field engineering (FFE) [22]. In addition, we choose the quadratic initial phase as an initial condition to reduce speckle caused by optical vortices. A typical sliced focal filed intensity profile generated by 3D focal field engineering is presented in Figure 1c. When exposing, we continuously switch these slices through uploading the phase masks onto the SLM and moving the XYZ-stage. Limited by the time response of the SLM, the switching speed is set to 30 phase masks per second. According to the thickness of the slices and the switching speed of the SLM, the velocity of the XYZ-stage is set to be 6 µm/s. It only takes 6.7 s to fabricate a Fresnel lens.

## 3. Results

**Figure 3:**
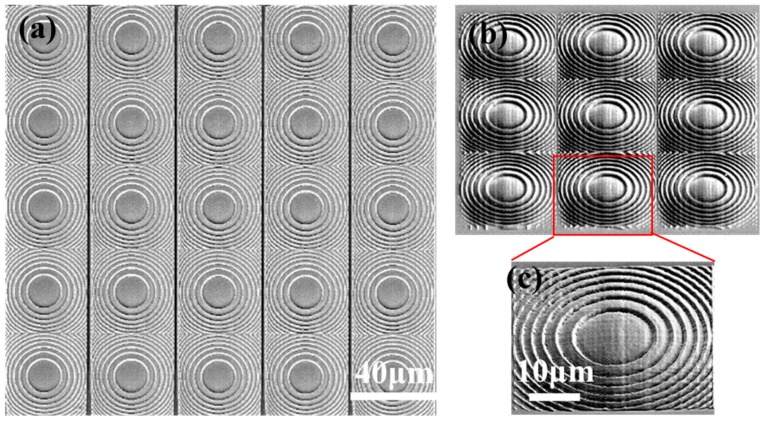
Continuous surface Fresnel lens array fabricated by femtosecond laser TPP via 3D focal field engineering. (**a**) The top view scanning electron microscopic (SEM) image of a 5 × 5 lens array; (**b**) The 45° side view SEM image of a 3 × 3 lens array; (**c**) The magnified SEM image of a lens in the 3 × 3 lens array.

The continuous surface Fresnel lens is fabricated by femtosecond laser TPP via 1D scanning using the 3D focal field engineering with rapid fabrication speed, so it is convenient to polymerize a Fresnel lens array. Figure 3 are the top view scanning electron microscopic (SEM) image of a 5 × 5 lens array (Figure 3a) and the 45° side view of a 3 × 3 lens array (Figure 3b), respectively. Each lens as shown in Figure 3c is polymerized with 1D scan within 7 s at the power of 300 mW. The surface of the lens is relatively smooth, showing the feasibility of this rapid fabrication technique.

**Figure 4:**
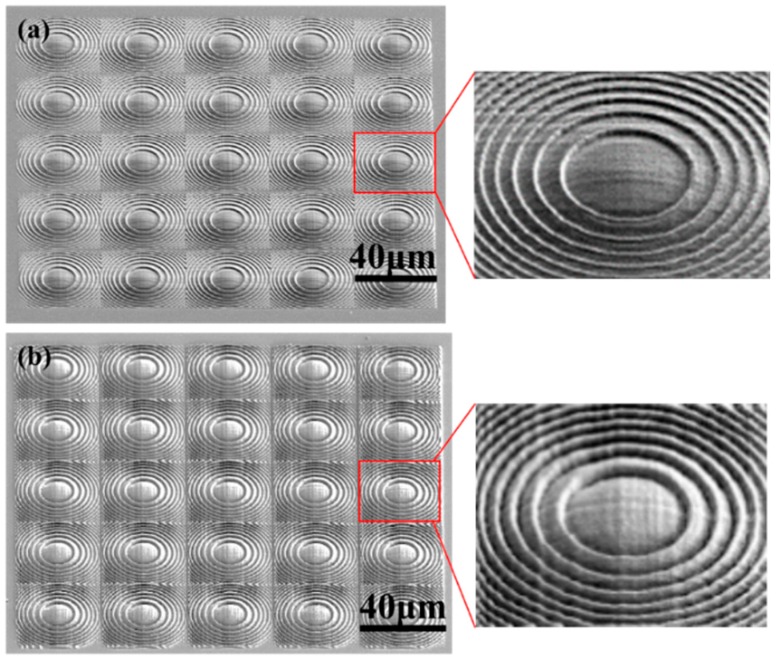
The 45° side view SEM image of the lens array annealed at (**a**) 120 °C and (**b**) 130 °C, respectively. The inset shows the magnified image of one lens in the array.

In order to further smoothen the surface of the lens, an effective and common thermal reflow strategy [28,29] is adopted. As demonstrated in Ref. [29], the polymerized structure can be further changed and reduced in size by high temperature treatment after the femtosecond laser induced polymerization. Therefore, we anneal lens arrays at 120 °C (Figure 4a) and 130 °C (Figure 4b) for 1 h, respectively, as post heat treatment. As a consequence, the surface smoothness is really improved as shown in the SEM images.

**Figure 5:**
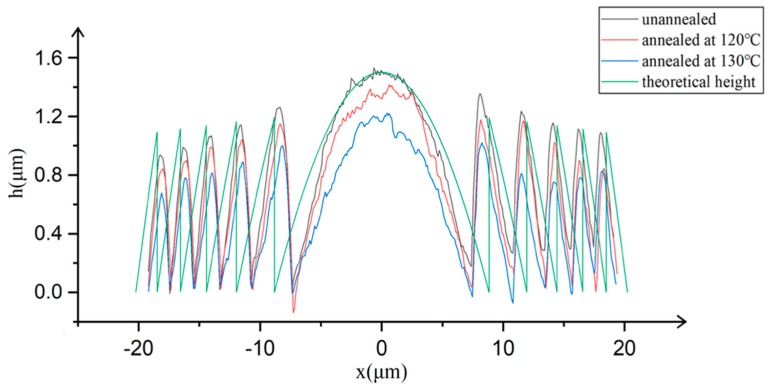
Surface profiles along the diameters of the Fresnel lenses. The green curve is the theoretical value of the desired Fresnel lens. The black, red and blue curves are results of the Fresnel lenses unannealed, annealed at 120 °C and 130 °C, respectively.

We measure the surface profiles along the diameters of the Fresnel lenses by the atomic force microscope (AFM). The results are shown in Figure 5 for the unannealed (black), annealed at 120 °C (red) and 130 °C (blue). The theoretical height (green) of the desired Fresnel lens is also presented for comparison. The real heights of the polymerized lens are near the theoretical values because we increase the height of the lens by 10% in advance to precompensate the height shrinkage. However, there exists 10% transverse shrinkage without precompensation. Estimated by NanoScope Analysis, the surface roughness (the average value of $R_{a}/R_{q}$) of microlenses for unannealed, annealed at 120 °C and 130 °C is 0.849, 0.838 and 0.771, respectively. The annealing really smoothens the surface but decreases the height too. Annealing at higher temperature (>130 °C) would distinctly reduce the heights of the polymer lenses to cause obvious height deviation.

**Figure 6:**
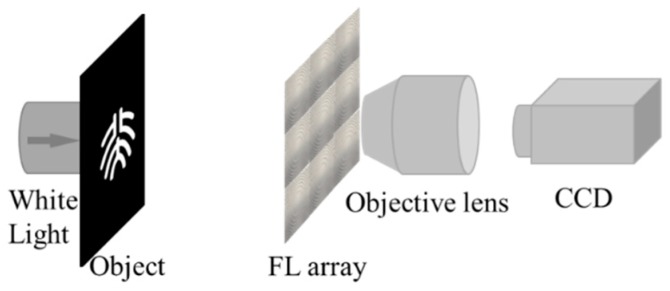
Schematic of the characterization system for the optical performance of the Fresnel lens array.

To characterize the focusing and imaging performance of the Fresnel lens array, an optical system is setup as shown in Figure 6. A white light beam is incident on the object such as the logo of Peking University before the Fresnel lens array. The object distance is much longer than the focal length of the Fresnel lens. Then, we capture the images by a CCD via a 60× objective lens.

**Figure 7:**
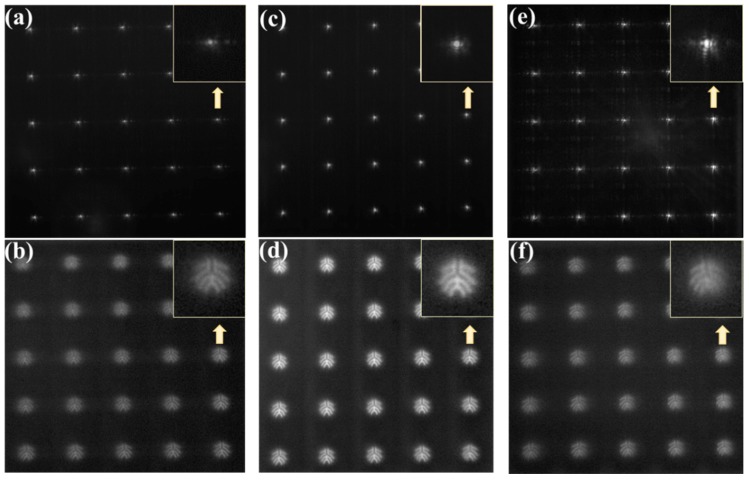
(**a**) Foci of the unannealed 5 × 5 lens array; (**b**) The image of the logo from the unannealed 5 × 5 lens array; (**c**) Foci of the 5 × 5 lens array annealed at 120 °C; (**d**) The image of the logo from the 5 × 5 lens array annealed at 120 °C; (**e**) Foci of the 5 × 5 lens array annealed at 130 °C; (**f**) The image of the logo from the 5 × 5 lens array annealed at 130 °C. The insets show the enlarged one of the images indicated by the arrows.

Without the object, the white light is directly incident on the 5 × 5 Fresnel microlens array to generate the focal spots with high quality as shown in Figure 7a. The 5 × 5 spots are almost the same, resulting from the high repeatability of this fabrication technique. The inset presents the enlarged image of the spot indicated by the yellow arrow. Figure 7b shows the images by the Fresnel lens array when the object is the logo of Peking University as demonstrated in Figure 6. From the enlarged image of one of the 5 × 5 images, we can see that imaging performance is very good.

As shown in Figure 7c,d, annealing at 120 °C for 1 h definitely enhances the focusing and imaging performance of the Fresnel microlens array by improving the surface smoothness after the heat treatment [28]. In addition, the contrast of the images is higher than that of the unannealed lens. However, the lens array annealed at 130 °C show noncircular foci (Figure 7e) and lower contrast images (Figure 7f) than those annealed at 120 °C. Although the lens has best smooth surface, the images are really blurred. It may be caused by the height discrepancy from the designed values after the overtreated annealing because the performance of a Fresnel microlens is highly sensitive to the height profiles. Thus, the Fresnel lens annealed at 120 °C are well suited for focusing and imaging functions.

## 4. Discussion

To ensure the configuration fidelity of the Fresnel microlens, several approaches are proposed and carried out. During the FFE, we set the sampling interval of the focal field in the z direction to be 50 nm to avoid pixelated roughness on the microlens surface due to the low sampling rate. The surface can be smoother if this interval is further decreased. Considering the shrinkage of the resist after exposure, we increase the height of the lens by 10% to precompensate when we design the target structure. Moreover, a pedestal with a thickness of 2 μm is added beneath the Fresnel lens to compensate the surface fluctuation or the tilt of the substrate.

To precisely control the surface profile of the lens in the future, we can further improve the generated intensity profile and precompensate the transverse shrinkages.

## 5. Conclusions

In conclusion, the continuous surface Fresnel lens array has been fabricated by rapid femtosecond laser TPP via 3D focal field engineering. Each 3D microlens is fabricated by continuous TPP of the 2D slices of the whole structure with 1D scan of the corresponding 2D engineered intensity within 7 s, which pave the way for rapid polymerization of large-scale lens arrays. Moreover, the lens array annealed at 120 °C has a smoother surface and exhibits better focusing and imaging performance, providing an approach using the thermal reflow to improve the smoothness of the surface.
